# Supplementary material for: Identification of a Tumor Cell Associated Type I IFN Resistance Gene Expression Signature of Human Melanoma, the Components of Which Have a Predictive Potential for Immunotherapy
Source: Int J Mol Sci. 2022 Feb 28;23(5):2704. doi: 10.3390/ijms23052704 (PMC8911010; doi:10.3390/ijms23052704)
Supplement: Supplementary file 1 [file ijms-23-02704-s001.zip › Figure S1.pdf]

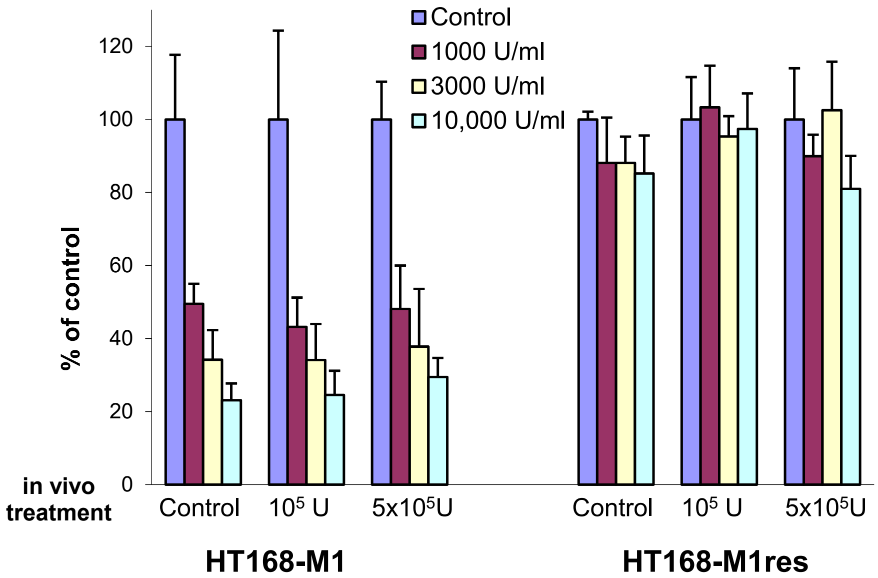

Figure S1. Effect of 5-day-long IFN-α2a treatment on the proliferation of HT168-M1 and HT168-M1res cells isolated from primary tumors of IFN-α2a-treated or control SCID mice (MTT assay, 5 parallel samples, mean ± SD)
